# The contribution of provitamin A biofortified cassava to vitamin A intake in Nigerian pre-schoolchildren

**DOI:** 10.1017/S0007114521000039

**Published:** 2021-11-14

**Authors:** Ibukun Afolami, Folake Samuel, Karin Borgonjen-van den Berg, Martin N. Mwangi, Olatundun Kalejaiye, Rasaki A. Sanusi, Linda Ayu Rizka Putri, Francesca Brivio, Inge D. Brouwer, Alida Melse-Boonstra

**Affiliations:** 1 Division of Human Nutrition and Health, Wageningen University & Research, Wageningen, the Netherlands; 2 Department of Human Nutrition, College of Medicine, University of Ibadan, Ibadan, Nigeria; 3 HarvestPlus Nigeria, c/o International Institute of Tropical Agriculture (IITA), Ibadan, Nigeria

**Keywords:** Biofortification, Yellow cassava, Vitamin A, Nigerian children

## Abstract

Biofortified yellow cassava has been developed to alleviate vitamin A deficiency. We examined the potential contribution of yellow cassava to total retinol activity equivalent (RAE) intake if replacing white by yellow cassava among pre-school Nigerian children. Dietary intake was assessed as part of a randomised controlled trial. Pre-schoolchildren (*n* 176) were randomly assigned to receive either white cassava (WC) or yellow cassava (YC) for 17 weeks. Dietary intake assessments were conducted during the intervention and 1 month after, when children had resumed their habitual diet. Differences in RAE intake between groups and time points were compared using a linear mixed model regression analysis. During intervention, median RAE intake was 536 µg/d in the YC group and 301 µg/d in the WC group (*P* < 0·0001). YC contributed approximately 40 % to total RAE intake. Of the children, 9 % in the YC group and 29 % in the WC group had RAE intake below the Estimated Average Requirement. After intervention, median RAE intake was 300 µg/d and did not differ between intervention groups (*P* = 0·5). The interaction effect of group and time showed a 37 % decrease in RAE intake in the YC group after the intervention (Exp(*β*) = 0·63; 95 % CI 0·56, 0·72). If WC was replaced by YC after intervention, the potential contribution of YC to total RAE intake was estimated to be approximately 32 %. YC increased total RAE intake and showed a substantially lower inadequacy of intake. It is therefore recommended as a good source of provitamin A in cassava-consuming regions.

Vitamin A deficiency (VAD) is still a public health problem in many low-to-middle income countries, affecting approximately 190 million pre-school age children, which corresponds to about 33 % of the children in this age group globally^(1)^. In 2013, the highest prevalence of VAD was in south Asia and sub-Saharan Africa with prevalence of 44 % and 48 %, respectively^(2)^. VAD impairs various physiological functions and, as a result, can pose serious health challenges to infants, children and pregnant women^(3)^. Approximately 94 500 deaths from diarrhoea and 11 200 deaths from measles worldwide were attributed to VAD in 2013, which accounted for 1·7 % of all deaths in children under 5 years in low-to-middle income countries^(1)^.

Nigeria is among the countries with the highest prevalence of VAD in Africa. Based on the available data from the only nationally representative food consumption and nutrient survey (2004), 29·5 % of children under 5 years old were classified as vitamin A deficient, with serum retinol concentrations below 0·7 μmol/l^(4)^, which is attributed to inadequate dietary intake. This high prevalence, which was regarded as a public health problem, led to the implementation of several strategies such as vitamin A supplementation and food fortification to reduce VAD prevalence^(5)^. Over the years, the coverage of vitamin A supplementation has increased in many low- to middle-income countries including Nigeria; however, the impact of vitamin A supplementation on the reduction of VAD has been at a very slow rate^(6)^.

A food-based approach is a more sustainable approach to attaining micronutrient adequacy compared with other approaches^(7)^. Biofortification has emerged as a complementary strategy to meet the micronutrient needs of vulnerable populations^(8)^. Specifically, several staples from sub-Saharan Africa and south Asia have been biofortified with provitamin A, with the aim of complementing other vitamin A interventions^(9)^. Two randomised controlled trials (RCT) have demonstrated the efficacy of provitamin A biofortified crops in increasing serum retinol^(10)^ and total body retinol pools^(11,12)^ in children. Yellow cassava, a biofortified variety of the traditional white cassava, is a root and tuber crop, largely consumed in Nigeria. There are currently six varieties of yellow cassava available in Nigeria. These varieties are generally resistant to many pests and diseases, have high yields and can produce up to 15 µg/g of *β*-carotene^(13)^. However, till now, no study has considered the contribution of yellow cassava to the usual intake of vitamin A in a natural setting, outside an experimental study set-up.

This study therefore aimed at quantifying the contribution of biofortified (yellow) cassava to vitamin A intake of pre-schoolchildren in a rural Nigerian community where cassava is widely consumed as a common staple, both under experimental and free-living conditions.

## Subjects and methods

### Study design and participants

Dietary assessment was conducted in 2016 as part of a RCT aimed at assessing the efficacy of biofortified cassava varieties on serum vitamin A concentrations in pre-schoolchildren, aged 3–5 years old in three adjacent communities: *Telemu*, *Ilemowu* and *Asamu* Osun state, Nigeria. The study was conducted according to the guidelines laid down in the Declaration of Helsinki, and all procedures involving human subjects were approved by the University of Ibadan/University College Hospital Ethical Review Committee, as well as a positive advice from the Medical Research Ethics Committee, Wageningen University and Research, Wageningen, the Netherlands. State Government approval was also obtained from the Osun State Ministry of Health. The study is registered in clinicaltrial.gov (ID NCT02627222). A total of 176 children were recruited into the RCT including dietary assessments. At least one participant was recruited from the twenty compounds identified in the communities, based on the following criteria: (1) their willingness to participate; (2) no visible sign of sickness; (3) older than 3 years and <5 years before study commencement; (4) plasma Hb ≥70 g/l and (5) ability to consume ≥80 % of the pre-established age-specific target meal portion for the RCT. Exclusion criteria included (1) severe anaemia (Hb<70 g/l)/symptomatic malaria or infectious diseases; (2) unwillingness to participate or no informed consent; (3) absenteeism (i.e. not meeting up to 20 % of feeding sessions); (4) inability to consume the required amount of cassava (i.e. ≥ 80 % of the age-specified amount) and (5) history of food allergy.

A pre-school was established specifically for the purpose of the intervention, where students were taught and fed between 08.00 and 14.00 hours. Children whose parents/guardian had signed a written informed consent and who met the conditions for the inclusion criteria were registered in the pre-school, where they were fed breakfast and lunch, 6 d/week, for a period of 17 weeks. The participants were divided into two groups: the experimental group (*n* 88) and the control group (*n* 88). The experimental group consumed foods prepared with yellow cassava (yellow cassava *eba*, yellow cassava *garri*, yellow cassava *moinmoin*, complemented with *okra* or *ewedu* soup), while the control group consumed the same foods prepared with white cassava. Children in the experimental and control groups were physically separated to prevent crossing-over. The children resumed school at 08.00 hours. Standardised recipes were developed for all foods consumed in the pre-school before the commencement of the intervention.

### Dietary intake assessments

No dietary assessment was conducted at baseline. The first round of dietary assessment was conducted in the 15th week of the study period (*n* 162) to estimate dietary intake of the experimental and control groups both within the pre-school and at home. A second round of dietary assessment (*n* 158) was conducted 1 month after the intervention, to measure the intake of the study population after they returned to their habitual diets. In each round, repeated recalls were conducted for approximately 30 % (*n* 45) of the children, to account for day-to-day variation and compute adjusted nutrient intake.

Within the pre-school, intervention foods were prepared in a central kitchen by trained cooks. All the ingredients used in cooking were weighed before cooking. Weights and total volume of dishes cooked were recorded daily and entered into a database. In the pre-school, trained research assistants weighed individual food portions before serving, using a 0·1 precision scale (Kern EMB 5.2K1). The same scales were used throughout the study period. All plates and cups were properly labelled with each participant’s ID number. After the meal, leftovers were recorded and entered the same day into a central database.

Dietary intake for foods consumed outside the pre-school was assessed using the quantitative interactive multi-pass 24-h recall method^(14,15)^ on evenly divided days over the week. Repeated recalls were conducted on non-consecutive days and distributed equally across the days of the week. Participants for repeated recalls were randomly selected from both experimental and control groups. Participants’ dietary consumption was provided by parents (mother or father) or caregivers, who were directly responsible for children’s diets. Dietary assessment was conducted at participant’s residence by local research assistants, who spoke the local language and had been trained shortly before the time of the dietary assessment.

During the interview, each parent/caregiver was asked to mention all foods and beverages consumed by their child (from waking up) on the day before the interview, until waking up on the interview day. Detailed information on food ingredients, including preparation/processing methods, total amount prepared, portion consumed by the child and amount of leftovers, were also collected during the recall session. Parents/caregivers were asked to demonstrate the procedures followed while preparing the recalled meals, using when possible, the same ingredients and household utensils (i.e. cups, bowls and spoons). From these demonstrations, the amount of each ingredient in each meal consumed was estimated by direct weighing on a digital scale (Kern EMB 5.2K1).

### Conversion factors

In households where the ingredients used for cooking were not available at the time of the recall, the volume or weight of comparable ingredients with similar texture was used as alternatives (i.e. proxy ingredients) during the demonstration, to estimate amount consumed (e.g. yam flour as an alternative to cassava flour, *garri* as an alternative to rice, etc.). After the recall, the actual ingredients were purchased from the community markets, and conversion factors (i.e. weight-to-weight or volume-to-weight) were computed, to calculate the actual amount of the recalled ingredient. In some households, some of the recalled meals were bought as ready-to-eat dishes from local food vendors. In other households, some of the ingredients used to prepare a particular meal were purchased from the community markets. In both cases, price-to-weight conversion factors were used to estimate the amount of ingredients consumed.

Most of the dishes reported during the recall session were prepared for the entire household, from which the child consumed only a portion. We therefore estimated the total volume of dish cooked and the proportion consumed by the child using the method described by Gibson & Ferguson^(14)^. Market surveys were conducted during and after the intervention to obtain all conversion factors. The market survey was conducted within 3 weeks after each 24-h recall data collection, to avoid the effect of price fluctuations on conversion factors. During the market surveys, food items or ingredients were purchased in the local currency (naira), from three different shops or vendors where community members frequently purchased their foods. The food items or ingredients were subsequently weighed with and without waste and then averaged. Price-to-weight conversion factors were generated for the edible portion of the food or ingredient, by calculating the amount in the local currency corresponding to 1 g of the food or ingredient. Weight-to-weight conversion factors were generated by calculating the amount of proxy ingredient corresponding to 1 g of actual ingredient. Volume-to-weight conversion factors were generated by calculating the volume of liquid/semi-solid food or ingredient corresponding to 1 g of the food or ingredient. During recall, it was noted that some dishes were prepared by combining various food items for a total price, for instance, 100 g rice + 50 g beans + 20 g stew = N50. In such cases, the proportion of each ingredient within the dish was calculated, so that the weight of each food item could be retrieved separately

### Standard recipes

During the 24-h dietary recall, the details of some food ingredients consumed by children could not be obtained because the dishes were bought from a food vendor or the respondent could not remember the ingredients in the dish. To address these issues, standard recipes were developed to estimate the amount of the ingredient consumed. To develop the standard recipes, three randomly selected volunteer mothers, whose children were participating in the study, were invited and asked to cook the specific dishes, using the same cooking methods employed at home. The different ingredients used in the preparation of the dishes were then recorded and weighed by research assistants while cooking was ongoing. Similarly, standard recipes were collected from random stalls and vendors within the community. In all cases, the edible portions of cleaned ingredients and the total volume of dish cooked were noted.

### Carotenoid analysis of food samples

During the entire study, samples of intervention meals, as consumed, were randomly collected twice weekly for 17 weeks, into opaque containers and homogenised with 5 ml butylhydroquinone (a preservative). Food samples were subsequently stored at −20°C at the field site and later shipped to Wageningen University and Research, where samples were stored at −80°C until analysis. During analysis, the food samples were pooled together as composite samples, representing three different intervention periods. Total *β*-carotene in food samples were analysed in duplicate using the HPLC (Thermo Scientific Accela LC system; Thermo Fisher Scientific) and EZCHrom Elite version 3.2.2 SP2 software (Argilent Technologies). The details of the extraction and HPLC analysis have been described elsewhere^(16)^.

### *β*-Carotene retention in commonly consumed palm oil soups

*β*-Carotene retention in two commonly consumed palm oil-based soups was calculated experimentally. Through the careful observation of volunteer mothers invited to cook these soups, a recipe detailing the average amount of all ingredients in the soup was developed and standardised, taking note of the average cooking time and bleaching temperatures for palm oil. The total *β*-carotene content of all raw ingredients was then calculated using a food composition table (FCT)^(17)^, by summing the *β*-carotene content of individual raw ingredients, including raw palm oil, which was obtained from the same community. In addition, the *β*-carotene content of raw palm oil was analysed. Using the standardised recipes, dishes of stew and *egusi* soups were prepared experimentally using either raw palm oil or standard zero carotenoid vegetable oil as ingredient. The quantity and type of all ingredients in the analysed soups were identical, and the only difference was the substitution of red palm oil (RPO) with zero carotenoid vegetable oil. Yield factors were calculated for both soups immediately after cooking, and the soups were analysed for total *β*-carotene content in the form normally consumed. The true retention factor of *β*-carotene (*βcar*) from the palm oil soups was estimated using the formula:
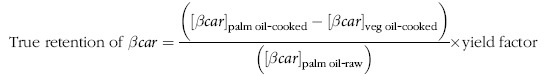
where [*βcar*]_palm oil-cooked_ = *β*-carotene content per g of cooked soup with palm oil; [*βcar*]_veg oil-cooked_ = *β*-carotene content per g of cooked soup with regular vegetable oil; and [*βcar*]_palm oil-raw_ = *β*-carotene content per g of palm oil in raw soup^(18)^.

### Nutrient intake estimation

To estimate nutrient intakes, a FCT was compiled for this study based on the Nutrient Composition of Commonly Eaten Foods in Nigeria – raw, processed and prepared^(19)^. The FCT was supplemented with nutrient composition from foods in other FCT in descending order of priority: West African Food Composition Table^(17)^; Condensed Food Composition Table for South Africa^(20)^; United States Department of Agriculture National Nutrient Database for Standard Reference, release 28^(21)^ and International Minilist^(22)^.

In the absence of nutrient information for particular foods, nutrient composition was obtained from nutrition labels (for packaged foods) or from published scientific literature. A bioequivalence factor of 7:1^(23)^ was applied to convert *β*-carotene to retinol activity equivalent (RAE) from cassava. For all other foods, a bioequivalence factor of 12:1 was applied^(24)^. Appropriate retention factors were applied to adjust for nutrient loss during preparation^(18)^. Compl-Eat software (version 1.0, Wageningen University and Research) was used in the overall computation of nutrient intake^(25)^. Vitamin A intake was expressed as RAE in μg/child per d. The average RAE intake was compared with the estimated average requirement (EAR) to determine the adequacy of vitamin A intake using age-specific cut-off points provided by the Institute of Medicine^(24)^. Estimations of RAE and percentage below EAR during the second round of dietary intake assessment were simulated, based on the assumption that the white cassava foods consumed during this time were replaced by yellow cassava foods. Total energy intake was expressed as kJ/child per d. Total energy intake was assessed by comparing total daily energy with the Estimated Energy Requirement as established by Institute of Medicine, which considers the sex, age, height, weight and physical activity levels. Based on the observation of the daily routine of the children, a physical activity level of 1·16 (low-active physical activity level) was used for all children to estimate their energy requirement^(24)^.

### Anthropometric measurement

Height and weight measurements were collected at baseline and endline, using a combined anthropometric 0·2 precision scale and stadiometer (Seca model no. 887 7021094), respectively. Anthropometric parameters (height-for-age, weight-for-age and weight-for-height *z*-scores) were analysed with Anthro (WHO version 3.2.2).

### Statistical analysis and sample size

The statistical analysis was carried out using Stata 13.0. The distribution of all variables used in the analyses was inspected for normality by combining QQ plots and visual inspections. Non-normal variables were log-transformed. All outliers were retained in the data because none of the estimated intake was considered implausible. Repeated recalls during each round of dietary intake assessment were analysed using ANOVA to estimate the magnitude of the within-person variance and to adjust for day-to-day variation. Following this procedure, adjusted intake distributions were calculated for RAE and energy intake, using the method described by the National Research Council^(26)^. A linear mixed regression method was used to estimate the difference (exp *β*) in RAE intake between the experimental and control groups based on the first and second rounds of dietary intake assessments. This model is advantageous because of its robustness in handling within-subject correlations resulting from repeated measurements. In fitting the model, log-transformed vitamin A intake was treated as the response variable and the intervention group was included as a fixed effect. Correlation between the two time points of dietary intake assessment was accounted for, by including the random intercept of the subject (ID) in the model. Unstructured covariance with restricted maximum likelihood estimation was used to estimate the variance component. Tukey–Kramer was applied to make multiple comparisons (interactions) of least square means among groups and time points. We adjusted for the confounding effect of age, sex and total energy intake by including these variables in the models. The best model was chosen by comparing the lowest Akaike information criterion. Wilcoxon non-parametric statistic was used to test for the difference in median energy and RAE between participants of different age categories.

Based on the projected daily intake of yellow and white cassava, we expected to detect a difference of at least 200 µg in the mean RAE daily intake between the yellow and white cassava groups. To this effect, a minimum sample size of 112 participants (*n* 56 per group) was required, assuming a 15 % attrition rate, an *α* and *β* risk of 0·05 and 0·20, respectively. This was based on a sd of 318 µg as reported in a study conducted in the same region^(27)^.

## Results

### Characteristics of the study population

A total of 162 children participated in the first round of the dietary intake assessment (Table 1), while 158 children participated in the second round (Table 2). The mean age of children was 4·1 (sd 1·0) years. Of the children, 58 % (*n* 94) of the children were males (Table 1).

**Table 1.** Characteristics of study participants (Mean values and standard deviations; numbers and percentages)

			During intervention				After intervention			
	All children (*n* 162)		Yellow cassava (*n* 82)		White cassava (*n* 80)		Yellow cassava (*n* 78)		White cassava (*n* 80)	
Characteristic	Mean	sd	Mean	sd	Mean	sd	Mean	sd	Mean	sd
Female										
*n*	68		35		33		33		34	
%	42		43		41		42		43	
Age (years)	4·1	1·0	4·1	1·03	4·1	0·90	4·4	1·07	4·5	1·0
Weight (kg)	14·38	2·06	14·38	2·13	14·38	2·0	14·9	2·11	14·8	1·93
Height (cm)	98·17	7·56	97·97	7·65	98·36	7·52	101	7·3	101	7·7
Weight-for-height *z*-score	−0·44	0·84	−0·52	0·84	−0·35	0·83	−0·72	0·80	−0·57	0·96
Weight-for-age *z*-score	−1·08	0·96	−1·05	0·93	−1·10	0·99	−1·12	0·93	−1·21	0·94
Height-for-age *z*-score	−1·29	1·31	−1·19	1·26	−1·39	1·37	−1·08	1·24	−1·28	1·36

**Table 2.** Energy and vitamin A intake during and after the intervention (Numbers and percentages; median values and 25th, 75th percentiles)

	During intervention				After intervention							
	Yellow cassava		White cassava		Yellow cassava		White cassava		Total (*n* 158)		Projected intake*	
Variables	Median	25th, 75th percentiles	Median	25th, 75th percentiles	Median	25th, 75th percentiles	Median	25th, 75th percentiles	Median	25th, 75th percentiles	Median	25th, 75th percentiles
Energy intake												
Low†												
*n*	20		11		16		12		28			
%	24		14		21		15		18			
<4 years, kJ/d	6301^a^	5531–7791	6510^a^	5184–7719	5987^a^	5778–6381	6004^b^	5832–6268	6000	5778–6381		
≥4 years, kJ/d	6527^a^	5430–7021	6933^a^	6008–7506	6150^a^	5678–6364	6104^b^	5870–6372	6113	5824–6372		
Girls, kJ/d	6498^a^	5627–7050	6933^a^	6150–7719	6025^a^	5387–6548	6079^a^	5983–6309	6079	5879–6431		
Boys, kJ/d	6385^a^	5485–7138	6590^a^	5845–7502	6075^a^	5653–6305	6071^a^	5766–6372	6075	5661–6314		
RAE intake												
Inadequacy												
*n*	7		23		38		32		70			
%	9		29		49		40		44			
<4 years, µg/d	563^a^	374–623	293^b^	260–392	249^b^	178–649	370^b^	170–622	307	177–625	475	313–626
≥4 years, µg/d	469^a^	328–586	303^b^	246–388	263^b^	184–532	350^b^	186–526	292	186–526	561	358–1017
Girls, µg/d	533^a^	308–606	310^b^	260–462	261^b^	198–528	397^b^	223–698	373	198–584	531	383–922
Boys, µg/d	542^a^	350–614	288^b^	251–363	254^b^	178–555	300^b^	162–450	273	163–480	475	318–743

RAE, retinol activity equivalents; IOM, Institute of Medicine.

a,b Median values within a row with unlike superscript letters are statistically significantly different. Row letters compare both yellow and white cassava groups during and after the intervention.

* Calculated from post-intervention data at time point 2 based on the assumption that yellow cassava foods replaced all white cassava foods for all children.

† Low energy intake is defined as energy intake less than IOM’s recommended age-specific requirement.

### Energy intake and vitamin A intake

The energy and vitamin A intakes during and after the intervention (i.e. in the first and second rounds of dietary intake assessment) are presented in Table 2. In the first round of dietary intake assessment, overall median energy intake was 6627 kJ/d, while in the second round, median energy intake was 6071 kJ/d (*P* < 0·001). There was no difference in energy intake between boys and girls (*P* = 0·24). Over 80 % of the children had adequate energy intake during and after the intervention.

During the intervention, median RAE intake was 536 µg/d and 301 µg/d in the yellow cassava and white cassava groups, respectively (*P* < 0·0001). However, after the intervention, median RAE intake decreased to 257 µg/d in the yellow cassava group and increased to 353 µg/d in the white cassava groups, respectively (*P* = 0·5). In total, 82 % of the children had adequate RAE intake during the intervention, which declined to 56 % after the intervention (*P* < 0·0001) (Table 2). There was no significant difference in RAE intake between boys and girls during and after the intervention (*P* = 0·58 and *P* = 0·13, respectively). During the intervention, yellow cassava contributed approximately 40 % to the total RAE intake and the percentage of children with retinol activity intakes below the EAR was 9 % (*n* 7) and 29 % (*n* 23) in the yellow and white cassava groups, respectively. After the intervention, the percentage of children with retinol intakes below the EAR increased to 43 %. Based on the assumption that consumed white cassava foods were all replaced with yellow cassava, the projected contribution to total RAE intake after intervention was estimated to be approximately 32 % (Table 2). Palm oil was the second largest contributor to vitamin A intake. During the intervention, palm oil contributed to approximately 24 % and 21 % of RAE intakes in the yellow and white cassava groups, respectively (*P* = 0·6). After the intervention, the contribution of palm oil to RAE intake was approximately 35 %.

Retention of *β*-carotene from palm oil in the soups is presented in Table 3. Estimated *β*-carotene retention from palm oil in stew and *egusi* vegetable soups was 6·9 % and 6·6 %, respectively.

**Table 3.** Retention of total *β*-carotene in red palm oil (RPO)-based soups*

Dish name	Raw ingredients			Cooked dish			
	Name	Amount (g)	*β*-Carotene† (μg)	Amount (g)	Yield factor	*β*-Carotene with RPO‡ (μg/100 g)	*β*-Carotene without RPO† (μg/100 g)
Stew	Tatase	69	1587	–		–	1746
	Chili pepper	39	250	–		–	244
	Onion	62	–	–		–	–
	Tomato	183	1142	–		–	1405
	Palm oil	200	137 360	–		–	–
	Seasoning	4	–	–		–	–
	Salt	2	–	–		–	–
	Water	250	–	–		–	–
	Total	809	140 761	370	0·46	5946	3395
*Egusi* soup	Tatase	108	2484				2732
	Chili pepper	78	499				488
	Onion	96	–				–
	Palm oil	250	171 700			–	
	Amaranthus	200	5780				5480
	Melon	60	–				–
	Salt	10	–				–
	Water	550	–				–
	Total	1352	180 463	935	0·69	9908	8700

* The carotenoid content of RPO is based on analysed value.

† Values are based on the reported values in the West-African Food Composition Table^(17)^.

‡ Values are based on laboratory analyses of cooked dish.

**Fig. 1.**
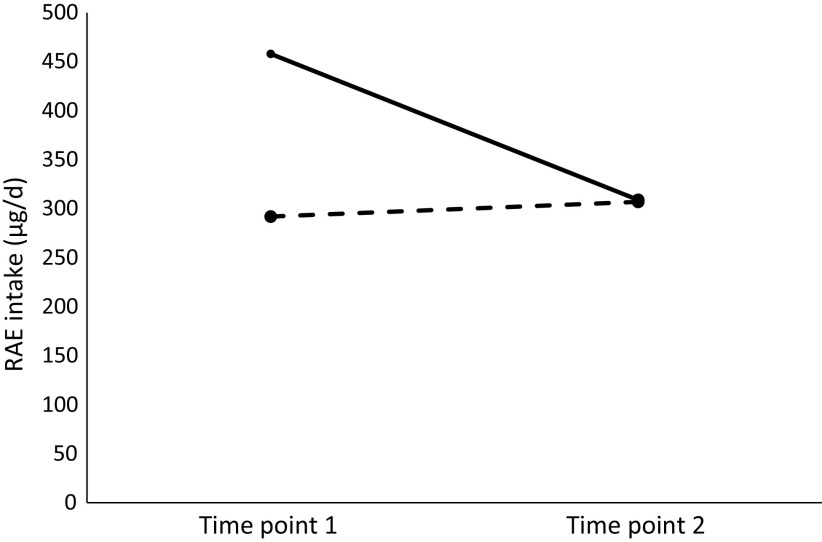
Estimated marginal mean retinol activity equivalent (RAE) intake in the yellow cassava and white cassava groups. Time point 1 = 1st round of dietary intake assessment (during intervention); time point 2 = 2nd round of dietary intake assessment (after intervention). 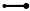, Yellow cassava; 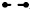, white cassava.

Table 4 presents the effect of yellow cassava on vitamin A intake after adjusting for energy intake, age and sex. Both treatment group (*P* < 0·0001) and time (*P* < 0·0001) significantly explained differences in RAE intake, and their interaction terms showed statistical significance as well. The main effect of the yellow cassava group *v*. the white cassava group on RAE intake was Exp(*β*) = 1·57 (95 % CI 1·43, 1·72), whereas the interaction effect of group and time showed that there was a 37 % decrease in RAE intake in the yellow cassava group after the intervention (Exp(*β*) = 0·63 (95 % CI 0·56, 0·72). Fig. 1 shows the estimated marginal mean intake of RAE in the yellow cassava and white cassava groups during and after the intervention: during the intervention, the yellow cassava and white cassava groups had mean intakes of 458 µg and 292 µg RAE, respectively, which declined to 309 µg in the yellow cassava group and increased to 307 µg in the white cassava group, after the intervention.

**Table 4.** Effect of yellow cassava on vitamin A intake (Mean values and 95 % confidence intervals)

Variable	*β* (exp)	95 % CI	*P* value
Intervention group*	1·57	1·43, 1·72	<0·0001
Time point†	1·06	0·97, 1·16	0·21
Group × time	0·63	0·56, 0·72	<0·0001

* Yellow cassava group *v*. white cassava group.

† Time point 2 (after intervention) *v*. time point 1 (during intervention).

## Discussion

This study aimed at estimating the contribution of pro-vitamin A biofortified (yellow) cassava to vitamin A intake in Nigerian pre-schoolchildren, both under experimental and free-living conditions. We found that vitamin A intake was 57 % higher in the yellow cassava group compared with the control group during the RCT, whereas intake declined after the intervention. During the RCT, yellow cassava provided sufficient RAE for nearly all the children (96 %). After the intervention, when children had returned to their habitual diets, replacement of white cassava with yellow cassava was estimated to still be able to provide for 32 % of total vitamin A intake and to reduce inadequacy of intake to 0 %.

The goal of pro-vitamin A biofortification programmes is to provide biofortified crops that will contribute at least 50 % of the EAR for provitamin A in target populations^(28)^. Our present findings show that the TMS 07/0593 variety of yellow cassava with approximately 9 µg/g (wet weight) *β*-carotene used in this study met the set goal under experimental conditions as well as when simulated under free-living conditions. Since breeding programmes are continuously ongoing, newly released varieties can be expected to perform even better. Our findings are also consistent, although higher than another study by Talsma *et al.* (2016), who, using a linear programming approach, showed that providing a school lunch with yellow cassava to children 6–12 years old could cover up to 47 % of the recommended nutrient intake of the schoolchildren^(10)^. Recommended nutrient intake are set as 2 sd above the EAR, hence the lower value in their study as compared with ours. Moreover, the methodological differences between the two studies could also be responsible for the differences in estimates.

An important contributor to vitamin A intake in the children’s diets was RPO. Over 80 % of the children consumed RPO at least once per d in one form or another. A different study around the same location showed that RPO consumption was ubiquitous and that about 43 % of children consumed RPO 6 times/week or more^(29)^. Raw RPO can contain high amounts of *β*-carotene and has been reported to range between 37 300 and 100 060 μg/100 g in West African diets^(17)^. To the best of our knowledge, the present study is the first to provide an estimate on *β*-carotene retention in commonly consumed Nigerian soups cooked with RPO, although there are few studies that have reported the total carotenoid and *β*-carotene content of these dishes^(30–33)^. However, the average retention of *β*-carotene in RPO estimated from this study may not be applicable to the entire Nigerian population, as cooking methods differ widely across ethnic groups in the country^(34)^.

RPO was mostly used to prepare stews and vegetable soups, which were usually consumed along with a starch-based staple food. A comparison of previous studies on the carotenoid content of *egusi* vegetable soup showed that *β*-carotene varied between 204 μg/100 g^(35)^ and 13 047 μg/100 g^(31)^. This large variation in carotenoid content can be partly explained by differences in cooking methods. For example, we observed that bleaching of RPO prior to cooking was a common practice amongst mothers in the community where our study was conducted. It may therefore be erroneous to apply the *β*-carotene concentration values of similar soups, directly from FCT without accounting for cooking time and method, and consequently, without appropriate retention factors for RPO, which is the largest source of carotenoids in these dishes^(32,36)^. We estimated that RPO consumption contributed about 35 % to total RAE intake, which was barely over half of the value reported by De Moura *et al*., in a study on cassava intake among women and pre-schoolchildren in Akwa-Ibom, southern Nigeria^(36)^. In that study, the contribution of palm oil to daily RAE was about three times higher than the estimated value in the present study. This disparity is probably because authors did not account for the loss of *β*-carotene from RPO upon heating since solid data for the *β*-carotene content of many Nigerian dishes are lacking. Based on the widespread consumption of RPO in Nigeria, further studies are needed to understand the amount of *β*-carotene that is retained from RPO-based foods under different cooking conditions. This will lead to more precise estimations of the contribution of RPO to vitamin A intake. Furthermore, it would also be interesting to explore the possibility of adapting cooking habits such that *β*-carotene from RPO is better retained.

From our study, despite the contribution of RPO, approximately 43 % of the children had inadequate vitamin A intake when consuming their habitual diets (Table 2). This clearly suggests that vitamin A intake is still lower than that recommended in this population, a gap which can be filled by promoting biofortification and other food-based strategies to improve vitamin A intake.

For the conversion of *β*-carotene to retinol, we assumed a bioequivalence factor of 7:1 for biofortified cassava-based foods in the present study. Some studies have shown that the bioequivalence of pro-vitamin A from biofortified cassava can be as high as 4:1 based on experimental studies with single meals under highly controlled conditions in healthy adults^(18)^. We used a more conservative value since the study was conducted in children living in a poor resource setting where malnutrition, intestinal parasite infestation and infectious disease are rife. If we would have used a higher conversion factor, the contribution of biofortified cassava to vitamin A intake would obviously have been even higher than our current estimates.

A very important strength of this study was that we measured nutrient intake during the intervention and afterwards, when the participants had returned to their usual diets. This provided a more accurate estimation of the contribution of yellow cassava to provitamin A intake during the intervention, as well as a realistic estimate of the potential increase in vitamin A intake that would occur if yellow cassava replaced white cassava. Other important strengths were the steps taken to improve the accuracy of nutrient intake estimation. First and foremost, we conducted laboratory analyses of biofortified food samples and commonly consumed RPO-based foods, which provided more reliable nutrient composition data, especially for biofortified foods which were not available in the national food composition database. Second, we developed recipes for all biofortified foods and commonly consumed local soups because standard recipes were unavailable for most of the soups consumed in the community households. Third, we estimated nutrient intake as a sum total of all individual ingredients used in the preparation of a particular dish. This method is deemed to be more reliable especially in rural communities in developing countries where standard recipes may not be available for many dishes^(37)^. Furthermore, we adjusted nutrient and energy intake distribution for day-to-day variation using repeated recalls. This provided a more accurate estimation of usual intake, by partially eliminating day-to-day variation.

This study also had some limitations. First, to estimate the nutrient value of some foods, values from FCT from multiple countries were adopted because the Nigerian Food Composition table did not cover the composition of all foods consumed in the community. A FCT in principle should be country-specific; thus, using FCT from other countries may have also introduced some errors in nutrient intake estimation. In addition, information on fortification was unavailable in most of the FCT consulted. Therefore, the vitamin A content of some fortified flour-based snacks, sugars and oils where proper nutrition labels were absent was not captured, thus contributing to errors. However, we expect that such errors would have been minimal because these food products were minimally consumed. For example, we were unable to compute possible vitamin A intake from possibly fortified flour used as an ingredient in biscuit, in which about 18 % of the children consumed an average of 23 g during the recall days. Moreover, compliance to mandatory food fortification for sugar and oil has been reported to be <20 % in Nigeria^(38)^. For our simulation, we assumed that, after intervention, all white cassava in the children’s diets would be replaced by biofortified varieties. However, in reality, this may not be the case, as some people may still have a high preference for white cassava. Finally, we did not account for seasonal variation in vitamin A intake. For example, certain pro-vitamin A-rich foods, such as mango, were unavailable during the season the study was conducted. This may also have contributed to errors in estimating the habitual intake of vitamin A as well as the percentage of children below the EAR.

In conclusion, yellow cassava contributed approximately 40 % of the total intake of vitamin A and has the potential to reduce the percentage of children at risk of inadequate intakes of vitamin A to a low level. Yellow cassava is therefore recommended as a good source of provitamin A in cassava-consuming regions.
